# Clinical, social, and occupational determinants of severe preeclampsia: a multifactorial case–control study on maternal health inequities in Peru

**DOI:** 10.1186/s12884-026-08653-w

**Published:** 2026-01-21

**Authors:** Jorge Ybaseta-Medina, Nicolasa Meza-León, Roberto Munive-Bendezú, Noemí Flores-Hernández, Luis Curotto-Palomino, Fermín Cáceres-Bellido, Víctor Barrientos-Ramos, Luciana Ybaseta-Soto

**Affiliations:** Facultad de Medicina Humana, Universidad Nacional San Luis Gonzaga, Ica, Perú

**Keywords:** Severe preeclampsia, Prenatal care, Occupational health, Psychosocial stress, Maternal morbidity, Peru, Case–control study

## Abstract

**Background:**

Severe preeclampsia remains a leading cause of maternal and perinatal morbidity and mortality, particularly in settings with limited access to quality antenatal care. In Peru, structural inequalities, physically demanding informal labor, and psychosocial stressors may exacerbate the risk of severe preeclampsia; however, local evidence integrating clinical and social determinants is scarce.

**Methods:**

An unmatched case–control study was conducted at a referral hospital in Ica, Peru, from 2018 to 2023. A total of 720 pregnant women were included: 237 cases with severe preeclampsia and 483 normotensive controls. Data were obtained exclusively from clinical records. Variables were grouped into six determinant domains (sociodemographic, occupational, antenatal access, obstetric history, nutritional status, and psychosocial factors), while perinatal outcomes were analyzed as secondary descriptive measures. Multivariable logistic regression was applied to identify independent factors associated with severe preeclampsia, focusing on etiological determinants rather than clinical prediction.

**Results:**

Severe preeclampsia was independently associated with a prior history of preeclampsia (adjusted OR = 5.40; 95% CI: 2.70–10.80), chronic hypertension (OR = 2.35; 95% CI: 1.45–3.82), obesity (OR = 2.05; 95% CI: 1.43–2.95), nulliparity (OR = 1.78; 95% CI: 1.28–2.47), fewer than four antenatal visits (OR = 1.69; 95% CI: 1.23–2.33), high physical workload (OR = 1.41; 95% CI: 1.01–1.96), psychosocial stress (OR = 1.83; 95% CI: 1.32–2.54), and family history of hypertension (OR = 1.47; 95% CI: 1.01–2.14). The regression model showed acceptable overall fit (Hosmer–Lemeshow *p* = 0.42).

**Conclusions:**

This study underscores the multifactorial nature of severe preeclampsia by integrating clinical, occupational, and psychosocial factors associated with the condition. The findings emphasize etiologically relevant associated factors rather than prediction, supporting their relevance for antenatal screening in contexts characterized by labor informality and limited obstetric coverage. These results support socially responsive preventive strategies emphasizing early and comprehensive prenatal care for vulnerable populations.

**Supplementary Information:**

The online version contains supplementary material available at 10.1186/s12884-026-08653-w.

## Introduction

Preeclampsia (PE) is a multisystem hypertensive disorder affecting 2–8% of pregnancies worldwide and remains a leading cause of maternal and perinatal morbidity and mortality, particularly in settings with limited antenatal care [1–3]. Its severe form, characterized by marked hypertension, proteinuria, and multiorgan dysfunction, constitutes an obstetric emergency with substantial risks for both mother and child [4–6].

In Latin America, hypertensive disorders of pregnancy account for up to 25% of maternal deaths [7]. In Peru, regions such as Ica illustrate the convergence of social and structural determinants that increase the risk of severe preeclampsia, including limited antenatal coverage, physically demanding work in non-regulated labor contexts, and geographic barriers to accessing healthcare services [8–10]. 

Classical risk factors such as nulliparity, obesity, chronic hypertension, and family history have been widely documented [11–14]. Emerging evidence also highlights the role of low socioeconomic status, anemia, adolescent pregnancy, and exposure to domestic violence in increasing PE risk [15–19]. Research from African contexts has further linked occupational stress and high physical workload to adverse obstetric outcomes, suggesting possible parallels in the Peruvian population [20–22]. In addition, recent studies have reported associations between psychosocial stress and preeclampsia, reinforcing the relevance of stress-related factors in maternal health [23–25].

Maternal vulnerability in Peru has additionally been associated with lack of partner support, preexisting anemia, and delays in timely medical care. PE is increasingly recognized as a clinical marker of future cardiovascular disease, underscoring the need for postpartum follow-up and long-term surveillance [5, 26].

This study aimed to generate local evidence on factors associated with severe PE among pregnant women receiving care at a referral hospital in Ica. Using an unmatched case–control design, we integrated conventional clinical variables with social, occupational, and psychosocial factors that remain underexplored in the national literature. The central hypothesis was that the interaction between established clinical antecedents [8–10], demanding occupational conditions [8, 9, 20, 22], limited antenatal access [27, 28], and prior psychosocial stress [29–31]—an area in which peer-reviewed evidence remains limited and heterogeneous—would be associated with a higher likelihood of severe PE. Recent meta-analyses support this hypothesis, reporting elevated odds ratios for a personal history of PE (OR = 5.6; 95% CI: 1.82–9.28), chronic hypertension, obesity, and inadequate antenatal care in vulnerable populations [32].

The findings were expected to strengthen high-risk antenatal screening and inform preventive strategies in regions characterized by extensive labor informality and limited obstetric coverage [27, 33]. Rather than proposing a predictive model, this study contributes clinically relevant evidence applicable to socially vulnerable environments, reinforcing the need for context-sensitive maternal surveillance.

## Methods

### Study design and setting

An analytical, unmatched case–control observational study was conducted in accordance with the Strengthening the Reporting of Observational Studies in Epidemiology (STROBE) guidelines for observational research [34]. The unmatched design was selected to maximize statistical efficiency and to allow the evaluation of multiple exposures without the constraints imposed by strict matching, which is particularly relevant for multifactorial outcomes such as severe preeclampsia (PE). This design enables robust estimation of associations between diverse exposures and a relatively uncommon outcome while optimizing the use of routinely collected clinical data in real-world healthcare settings.

The study was carried out at Hospital Santa María del Socorro, a public referral hospital in Ica, Peru, between January 2018 and December 2023. This institution manages approximately 2,200–2,300 deliveries per year and serves women from both urban and rural areas, including populations with limited access to specialized obstetric services.

### Participants and eligibility criteria

#### Case definition and diagnostic consistency across the study period (2018–2023)

Cases were identified through a standardized chart-abstraction protocol applied uniformly across all study years. For diagnostic coding, we used the Peruvian Ministry of Health (MINSA) *Clinical Practice Guideline for Hypertensive Disorders of Pregnancy* (2020) [26], while epidemiological classification followed the criteria of the American College of Obstetricians and Gynecologists (ACOG, 2020) and the International Society for the Study of Hypertension in Pregnancy (ISSHP) [4, 5].

Case determination did not rely exclusively on the clinician’s recorded diagnosis. Instead, two trained reviewers independently verified each case by reapplying contemporary ACOG/ISSHP diagnostic criteria [4, 5]. Verification required confirmation of severe hypertension (systolic blood pressure ≥ 160 mmHg or diastolic blood pressure ≥ 110 mmHg on two measurements) together with at least one marker of maternal organ dysfunction, including renal impairment, thrombocytopenia, hepatic dysfunction, pulmonary edema, or new-onset neurological symptoms. Evidence of organ dysfunction was extracted from institutional laboratory reports, nursing charts, and physician progress notes. Any discrepancies between reviewers were resolved by consensus with a senior obstetrician.

To ensure temporal consistency, identical abstraction rules and diagnostic thresholds were retrospectively applied to all records from 2018 to 2023, regardless of the terminology used at the time of clinical documentation. Although earlier national records employed the term “preeclampsia grave,” all cases were reclassified according to the current standardized terminology “preeclampsia with severe features,” which is used consistently throughout the manuscript [4, 5, 26].

Complete diagnostic criteria and exclusion parameters are provided in Supplementary File 1.

### Sample size and recruitment

Sample size was estimated assuming 90% statistical power, a two-sided α of 0.05, and an anticipated odds ratio of 8.0–9.0 for a personal history of preeclampsia—one of the strongest established predictors of recurrent disease according to meta-analytic evidence [32]. Based on a 1:2 case-to-control ratio and allowing for 15% attrition due to incomplete records, the minimum required sample was 237 cases and 483 controls. Cases were enrolled consecutively upon diagnosis confirmation, whereas controls were selected through simple random sampling stratified proportionally across the study period (2018–2023). The final sample size and case-to-control ratio align with those reported in comparable hospital-based case–control studies conducted in resource-limited referral settings [33].

### Data analysis

Statistical analyses were performed using SPSS version 28 and R version 4.3.1. Descriptive statistics summarized the study population: categorical variables were expressed as frequencies and percentages, and continuous variables were reported as mean ± standard deviation (SD) or median (interquartile range, IQR), according to their distribution. Group comparisons were conducted using the Chi-square or Fisher’s exact test for categorical variables and Student’s t test or Mann–Whitney U test for continuous variables, as appropriate.

Multivariable analysis followed an etiological framework aimed at identifying factors associated with severe preeclampsia rather than developing a clinical prediction model. Accordingly, predictive performance metrics were not evaluated; model fit was assessed using the Hosmer–Lemeshow goodness-of-fit test. A clinically informed set of confounders was prespecified based on prior literature and plausibility, including maternal age, education level, and place of residence. Additional variables were selected based on established associations with severe preeclampsia and their relevance within a causal framework, consistent with recommended approaches for confounder control in observational studies [39, 40]. All selected variables were entered simultaneously into a multivariable logistic regression model.

The variable “acute emotional overload” was excluded from multivariable analyses because its temporal proximity to the diagnosis of preeclampsia compromises causal inference. Prespecified interaction terms between obesity and number of antenatal visits, as well as between rural residence and geographic barriers, were explored based on prior evidence of effect modification in similar contexts [41].

The overall proportion of missing data was 8.5%, primarily due to incomplete clinical or occupational records. Because documentation patterns may reflect structural vulnerability, missing data were interpreted in light of evidence indicating that social determinants influence patterns of data completeness and cardiovascular outcomes in pregnancy, particularly among structurally vulnerable populations [42]. A complete-case analysis was conducted for the primary models, and multiple imputation using chained equations was performed as a sensitivity analysis to assess the robustness of the estimates [43].

### Ethical considerations

The study was reviewed and approved by the Institutional Research Ethics Committee of Hospital Santa María del Socorro (CIEI-HSMS), Ica, Peru (approval code CIEI HSMS 2024–069). The committee granted a waiver of written informed consent due to the retrospective nature of the study and the exclusive use of anonymized medical records. All procedures complied with the Declaration of Helsinki (2013 revision), CIOMS guidelines, and Peruvian national regulations.

## Results

### General description of the study population

Data from 720 pregnant women were analyzed, including 237 cases diagnosed with severe preeclampsia (PE) and 483 normotensive controls, maintaining an approximate 1:2 case–control ratio, which enhanced the statistical efficiency of the study design. Primary analyses were conducted using a complete-case approach; findings were materially unchanged in sensitivity analyses using multiple imputation (data not shown), supporting the robustness of the results.

### Sociodemographic characteristics

The mean maternal age was slightly higher among cases compared to controls (30.4 ± 6.2 vs. 27.9 ± 5.8 years), although this difference did not reach statistical significance (*p* = 0.080). Adolescent pregnancy (< 18 years) was observed in 7.6% of cases and 4.8% of controls (*p* = 0.123). In contrast, advanced maternal age (≥ 35 years) was significantly more frequent among cases (24.5% vs. 13.4%, *p* < 0.001).

Additional sociodemographic disparities were evident: single marital status (38.4% vs. 22.6%, *p* < 0.001), primary education level (29.1% vs. 17.4%, *p* < 0.001), rural residence (27.2% vs. 10.4%, *p* < 0.001), and low socioeconomic status (41.8% vs. 24.8%, *p* < 0.001) were all significantly more common among women with severe PE (Table 1).

**Table 1:** Sociodemographic characteristics of participants

Variable	Cases (*n* = 237)	Controls (*n* = 483)	Total (*n* = 720)	*p*-value
Age (years), mean ± SD	30.4 ± 6.2	27.9 ± 5.8	28.7 ± 6.0	0.080
Age < 18 years (adolescent)	18 (7.6%)	23 (4.8%)	41 (5.7%)	0.123
Age ≥ 35 years (advanced maternal age)	58 (24.5%)	65 (13.4%)	123 (17.1%)	< 0.001
Marital status: Single	91 (38.4%)	109 (22.6%)	200 (27.8%)	< 0.001
Education: Primary level	69 (29.1%)	84 (17.4%)	153 (21.3%)	< 0.001
Rural residence	65 (27.2%)	50 (10.4%)	115 (16.0%)	< 0.001
Low socioeconomic status	99 (41.8%)	120 (24.8%)	219 (30.4%)	< 0.001

Univariate analysis was performed using Pearson’s Chi-square test for categorical variables and Student’s t-test for continuous variables. Results are presented as absolute frequencies (n), percentages (%), and mean ± standard deviation (SD). Statistical significance was set at *p* < 0.05

### Occupational conditions

Women with severe PE were more frequently engaged in physically demanding and informal occupations. Compared with controls, they showed significantly higher proportions of fieldwork (23.6% vs. 12.8%, *p* < 0.001) and informal commerce (31.2% vs. 21.5%, *p* = 0.004). Occupational strain indicators were also more common among cases, including high physical workload (39.7% vs. 20.3%, *p* < 0.001) and workweeks exceeding 40 h (28.4% vs. 15.5%, *p* < 0.001) (Table 2).

**Table 2:** Occupational characteristics of participants

Variable	Cases (*n* = 237)	Controls (*n* = 483)	Total (*n* = 720)	*p*-value
Fieldwork, n (%)	56 (23.6%)	62 (12.8%)	118 (16.4%)	< 0.001
Informal commerce, n (%)	74 (31.2%)	104 (21.5%)	178 (24.7%)	0.004
High physical workload, n (%)	94 (39.7%)	98 (20.3%)	192 (26.7%)	< 0.001
Workweek > 40 h, n (%)	67 (28.4%)	75 (15.5%)	142 (19.7%)	< 0.001

Univariate comparisons were performed using Pearson’s Chi-square test. Results are presented as absolute frequencies (n) and percentages (%). Statistical significance was set at *p* < 0.05

### Psychosocial factors

Prior psychosocial stress was significantly more frequent among cases than controls (45.9% vs. 27.3%, *p* < 0.001). This finding indicates that documented emotional or social stressors were more commonly recorded among women with severe PE (Table 3).

**Table 3:** Psychosocial characteristics of participants

Variable	Cases (*n* = 237)	Controls (*n* = 483)	Total (*n* = 720)	*p*-value
Prior psychosocial stress	109 (45.9%)	132 (27.3%)	241 (33.5%)	< 0.001

Univariate comparisons were performed using Pearson’s Chi-square test. Results are presented as absolute frequencies (n) and percentages (%). Statistical significance was set at *p* < 0.05

### Access to antenatal care

Women with severe PE were less likely to have received adequate antenatal care (≥ 4 visits) and more likely to experience geographic barriers, including travel times > 30 min to a health facility or > 1 h to a referral hospital. The use of traditional medicine was also more frequent among cases (Table 4).

**Table 4:** Access to prenatal care

Variable	Cases (*n* = 237)	Controls (*n* = 483)	Total (*n* = 720)	*p*-value
< 4 prenatal visits	85 (35.9%)	90 (18.6%)	175 (24.3%)	< 0.001
Distance > 30 min to health facility	62 (26.2%)	58 (12.0%)	120 (16.7%)	< 0.001
Travel time > 1 h	43 (18.1%)	36 (7.4%)	79 (11.0%)	< 0.001
Use of traditional medicine	29 (12.2%)	30 (6.2%)	59 (8.2%)	0.007

Univariate analysis was performed using Pearson’s Chi-square test. Statistical significance was set at *p* < 0.05

### Obstetric and medical history

Several established clinical risk factors were significantly more frequent among cases than controls. Nulliparity was more common in women with severe PE (48.5% vs. 25.3%, *p* < 0.001). A prior history of preeclampsia showed a marked difference between groups (22.4% vs. 3.1%, *p* < 0.001), as did chronic hypertension (19.8% vs. 6.4%, *p* < 0.001). Cases also presented higher frequencies of gestational diabetes (11.4% vs. 4.8%, *p* = 0.001), urinary tract infection (16.0% vs. 9.3%, *p* = 0.009), and family history of hypertension (19.4% vs. 10.1%, *p* < 0.001).

### Nutritional status

Obesity and low intake of fruits and vegetables (< 3 servings/day) were significantly more common among cases, supporting the relevance of nutritional assessment in antenatal care (Table 5).

**Table 5:** Nutritional status of participants

Variable	Cases (*n* = 237)	Controls (*n* = 483)	Total (*n* = 720)	*p*-value
Obesity (based on clinical records)	97 (41.0%)	108 (22.4%)	205 (28.5%)	< 0.001
Low intake of fruits/vegetables	87 (36.7%)	94 (19.5%)	181 (25.1%)	< 0.001

Univariate analysis was performed using Pearson’s Chi-square test to compare proportions between groups. Statistical significance was set at *p* < 0.05

### Perinatal outcomes

Women with severe PE showed significantly higher frequencies of cesarean delivery, preterm birth, low neonatal weight, and adverse neonatal outcomes, highlighting marked differences in maternal and neonatal outcomes between groups.

### Multivariable model

The multivariable logistic regression identified several independent factors associated with severe PE. Significant associations were observed for history of PE (adjusted OR = 5.40; 95% CI: 2.70–10.80), chronic hypertension (adjusted OR = 2.35; 95% CI: 1.45–3.82), obesity (adjusted OR = 2.05; 95% CI: 1.43–2.95), nulliparity (adjusted OR = 1.78; 95% CI: 1.28–2.47), fewer than four prenatal visits (adjusted OR = 1.69; 95% CI: 1.23–2.33), high physical workload (adjusted OR = 1.41; 95% CI: 1.01–1.96), prior psychosocial stress (adjusted OR = 1.83; 95% CI: 1.31–2.57), and family history of hypertension (adjusted OR = 1.47; 95% CI: 1.01–2.14). The model demonstrated adequate fit (Hosmer–Lemeshow *p* = 0.42) (Table 6).

**Table 6:** Factors associated with severe preeclampsia (multivariable model)

Variable	Adjusted OR	95% CI	*p*-value
History of PE	5.40	2.70–10.80	< 0.001
Chronic hypertension	2.35	1.45–3.82	< 0.001
Obesity (based on clinical record)	2.05	1.43–2.95	< 0.001
Nulliparity	1.78	1.28–2.47	0.001
< 4 prenatal visits	1.69	1.23–2.33	0.001
High physical workload	1.41	1.01–1.96	0.044
Prior psychosocial stress	1.83	1.31–2.57	< 0.001
Family history of hypertension	1.47	1.01–2.14	0.043

Binary logistic regression model adjusted for prespecified confounders (maternal age, education, and residence) and additional clinically relevant covariates defined a priori in the analytical framework. Statistical significance was set at *p* < 0.05

## Discussion

This study identified multiple factors independently associated with severe preeclampsia (PE) by integrating clinical, social, occupational, and psychosocial variables within an etiological framework. Rather than proposing a predictive model, the findings underscore the multifactorial nature of severe PE in vulnerable settings, consistent with previous systematic reviews and observational evidence [1, 2, 44]. The multivariable logistic regression model demonstrated adequate fit (Hosmer–Lemeshow *p* = 0.42), supporting the internal coherence of the analytical approach. Importantly, perinatal outcomes were not conceptualized or analyzed as determinants of severe PE; instead, they were treated as downstream clinical consequences and reported descriptively to preserve temporal and causal coherence.

A prior history of PE showed the strongest association with severe PE (adjusted OR = 5.40), in line with meta-analyses reporting relative risks exceeding 8.0 [12, 44–47]. Chronic hypertension (OR = 2.35) and nulliparity (OR = 1.78) were also independently associated, confirming their consistent role as major risk factors across diverse populations [2, 48–50]. The potential interaction between nulliparity and obesity may reflect heightened maternal immune activation and endothelial vulnerability during first pregnancies, thereby increasing susceptibility to hypertensive disorders of pregnancy [51, 52].

Obesity (OR = 2.05) was associated with increased odds of severe PE, supporting extensive evidence linking elevated body mass index to endothelial dysfunction, systemic inflammation, and impaired placental angiogenesis [13, 47, 53]. Dietary factors, such as low intake of fruits and vegetables, were associated with severe PE in bivariate analyses but were not retained in the final multivariable model. This likely reflects confounding with socioeconomic status and obesity-related metabolic pathways, suggesting that nutritional intake may act through broader structural and nutritional status determinants rather than as an independent exposure.

Limited antenatal access (OR = 1.69), particularly among women with fewer than four prenatal visits, remained significantly associated with severe PE. This finding aligns with evidence from middle-income countries linking inadequate antenatal coverage to increased hypertensive morbidity [15, 54]. The protective role of antenatal care extends beyond visit frequency and is shaped by timing, quality, and structural barriers—including geographic distance, cultural or ethnic discordance between patients and providers, and psychosocial stressors—that may delay detection and management of hypertensive complications [16, 55–58].

High physical workload (OR = 1.41) was independently associated with severe PE, suggesting that sustained physical effort may function as a hemodynamic and metabolic stressor during pregnancy. Although the literature on occupational exposures and PE remains limited, studies conducted in rural and informal labor contexts—particularly agriculture and informal commerce—have reported comparable associations [20, 59, 60]. Other occupational characteristics, such as prolonged working hours and informal commerce, did not persist in the final model, likely due to confounding with broader structural determinants.

Prior psychosocial stress (OR = 1.83) remained significantly associated with severe PE after multivariable adjustment. Biological plausibility linking chronic stress to hypertensive disorders of pregnancy has been proposed through activation of the hypothalamic–pituitary–adrenal axis, endothelial dysfunction, and placental insufficiency [61–64]. However, the available literature remains limited, heterogeneous, and in several cases based on small or non–peer-reviewed studies, particularly in low- and middle-income countries. This scarcity of robust evidence underscores the relevance of our findings, which contribute clinically documented, real-world data from a vulnerable population. Although retrospective ascertainment may lead to underreporting, reliance on documentation recorded prior to diagnosis supports temporal validity and reduces recall bias. The evolving definition of PE—no longer requiring proteinuria and discouraging the use of the term “severe”—further supports the integration of psychosocial and contextual variables into early risk stratification frameworks [5, 26, 64].

A family history of hypertension (OR = 1.47) supports the hypothesis of genetic and epigenetic predisposition [65–67]. As a low-cost, high-specificity marker, this variable is particularly valuable in low-resource settings, facilitating early identification of high-risk pregnancies without reliance on advanced diagnostics. Its inclusion in screening algorithms may enhance surveillance among socially vulnerable populations [68, 69].

These findings parallel results from other low-resource settings, such as Sierra Leone, where obesity, limited antenatal access, and family history of hypertension were similarly associated with hypertensive disorders of pregnancy. In both contexts, structural vulnerability, informal labor, and psychosocial stress appear to shape risk patterns, underscoring the importance of incorporating social and economic context into maternal health strategies [8, 10, 20, 59]. By integrating occupational and psychosocial domains—rarely addressed in conventional analyses—this study broadens understanding of PE-related risk and supports more comprehensive, preventive, and context-sensitive approaches [45, 56, 59, 62, 70].

### Strengths and limitations of the study

This study has several strengths, including an adequate sample size with 90% statistical power, a multifactorial analytical approach integrating clinical and social determinants, and adjustment for key confounders informed by causal reasoning [33]. The use of real-world clinical records from a referral hospital enhances contextual validity, while rigorous statistical methods—including multiple imputation for missing data and prespecification of prior PE as an a priori variable—support internal validity [12, 15, 39, 40]. Adherence to STROBE guidelines further strengthens methodological transparency [34].

Several limitations warrant consideration. The retrospective design may have led to underdocumentation of psychosocial variables, although reliance on documented records reduces recall bias. Exclusion of women with severe comorbidities was necessary to reduce confounding but may limit generalizability. Incomplete documentation of informal employment patterns may have resulted in occupational exposure misclassification, potentially biasing estimates toward the null [55, 71]. Additionally, the single-region design may limit external validity, although the characteristics of Ica are representative of similar resource-limited settings in Peru and Latin America [8, 10]. Finally, severe maternal outcomes such as eclampsia, HELLP syndrome, and maternal death were not consistently documented, restricting the scope of outcome assessment.

#### Implications for practice and policy

The identification of modifiable associated factors—including psychosocial stress, high physical workload, obesity, and limited antenatal access—provides actionable targets for primary care and public health interventions in vulnerable populations. While nutritional intake may also represent a modifiable domain, its role in this study appears to be mediated by broader socioeconomic and nutritional status determinants rather than acting independently. Incorporating these factors into national protocols, such as the MINSA guideline for the prevention and management of preeclampsia and eclampsia [26] and aligning with recent FIGO recommendations on integrating social determinants into first-trimester screening [72], may strengthen early risk identification and targeted follow-up in low-resource settings.

Integrating social and clinical factors into routine antenatal care represents a critical step toward more equitable, context-sensitive maternal health strategies. Local evidence from the same region demonstrates that failure to address social and educational barriers—particularly delays in seeking care and lack of awareness of warning signs—is associated with preventable maternal deaths [10]. Thus, these findings reinforce the need for holistic models of care to prevent progression from severe morbidity to maternal mortality in vulnerable populations.

## Conclusions

This study identified multiple factors independently associated with severe preeclampsia by integrating conventional clinical risk factors with social, occupational, and psychosocial variables. History of preeclampsia, chronic hypertension, nulliparity, and obesity were confirmed as consistent associated factors, while limited antenatal access, high physical workload, and prior psychosocial stress provided novel evidence on underexplored domains in the Peruvian context.

The findings support a comprehensive approach to antenatal screening in settings characterized by labor informality and restricted access to maternal health services. Recognition of modifiable associated factors offers opportunities for equity-oriented preventive interventions in primary care, promoting risk-stratified and socially responsive antenatal care. Rather than presenting a predictive model, this study strengthens maternal surveillance strategies in regions with a high burden of hypertensive morbidity and contributes contextually relevant evidence to inform maternal health policy in Peru and similar Latin American settings. 

## Supplementary Information

Supplementary Material 1. [1–5, 9–15, 26, 29–31, 33, 35–38].

Supplementary Material 2.

Supplementary Material 3.

Supplementary Material 4.

## Data Availability

The datasets generated and analyzed during the current study are not publicly available due to institutional restrictions. Data are available from the corresponding author upon reasonable request and with permission from the Institutional Research Ethics Committee of Hospital Santa María del Socorro.
