# Supplementary material for: Clinical, social, and occupational determinants of severe preeclampsia: a multifactorial case–control study on maternal health inequities in Peru
Source: BMC Pregnancy Childbirth. 2026 Jan 21;26:297. doi: 10.1186/s12884-026-08653-w (PMC12998109; doi:10.1186/s12884-026-08653-w)
Supplement: Supplementary file 1 — Supplementary Material 1. [1–5, 9–15, 26, 29–31, 33, 35–38]. [file 12884_2026_8653_MOESM1_ESM.docx]

**Supplementary File 1**

**Operational Definitions and Methodological Details**.

**Definition of controls**

Controls were normotensive pregnant women delivering at the same institution during the study period, randomly selected from hospital birth records. Controls were required to have at least one documented blood pressure measurement during prenatal care or at admission and no evidence of gestational hypertension, preeclampsia, eclampsia, or significant proteinuria.

The same exclusion criteria were applied to cases and controls to minimize confounding by conditions independently associated with hypertensive disorders of pregnancy. These exclusions included multiple gestation, major fetal malformations, chronic kidney disease, pregestational diabetes mellitus, and systemic lupus erythematosus. Chronic hypertension was not an exclusion criterion, as it represents a well-established etiological risk factor for severe and superimposed preeclampsia and was therefore retained as an exposure variable.

**2.3 Data sources and exposure ascertainment**

All study variables were obtained exclusively from routinely collected clinical records, including standardized prenatal intake forms, obstetric admission notes, laboratory reports, nursing charts, and physician progress notes. Occupational information is routinely documented at the first prenatal visit and/or at hospital admission, while psychosocial conditions may be recorded during prenatal follow-up or through social work assessments when identified during care.

To reduce the risk of outcome-dependent documentation bias, exposure ascertainment for occupational, nutritional, and psychosocial variables was restricted, whenever possible, to information recorded prior to the diagnosis of preeclampsia among cases and prior to delivery among controls. Peri-diagnostic or post-diagnostic notes were not used to define antecedent exposures.

**2.4 Variable domains and operational definitions**

The dependent variable was the diagnosis of severe preeclampsia. Diagnostic coding followed the Peruvian Ministry of Health (MINSA) Guideline for Hypertensive Disorders of Pregnancy (2020), while epidemiological classification was aligned with the American College of Obstetricians and Gynecologists (ACOG, 2020) and International Society for the Study of Hypertension in Pregnancy (ISSHP) criteria [4,5,26]. Independent variables were grouped into seven domains: sociodemographic characteristics, occupational conditions, antenatal access, obstetric and medical history, nutritional status, psychosocial factors, and perinatal outcomes [1–5]. Maternal age, education level, and place of residence were prespecified as key confounders based on prior evidence and were included in multivariable models regardless of statistical significance.

**Sociodemographic variables**

Maternal age, education level, marital status, place of residence, and socioeconomic status were extracted from standardized prenatal intake forms and social work documentation. Adolescent pregnancy was defined as maternal age <18 years, and advanced maternal age as ≥35 years. Rural residence was assigned to women living in peripheral or rural areas with limited access to specialized obstetric services. Low socioeconomic status was defined using three routinely documented indicators: informal employment, lack of basic household services (water, sanitation, or electricity), and documented social vulnerability such as domestic violence, extreme poverty, or unstable housing. Women meeting at least two of these criteria were classified as having low socioeconomic status [12,15].

**Occupational variables**

Occupational variables included type of occupation, informal labor status, physical workload, and work intensity. Informal labor was defined as employment lacking a formal contract, social security coverage, or regulated employment benefits. High physical workload was characterized by sustained physical effort, prolonged standing for more than six hours per day, or continuous movement during work activities. Long work hours were defined as employment involving at least 40 hours per week during the first and second trimesters of pregnancy [7–9].

Occupational titles recorded in clinical charts were coded using the *Clasificación Nacional de Ocupaciones* (CNO–Perú) and subsequently mapped to the major groups of the *International Standard Classification of Occupations* (ISCO-08). Based on this mapping, occupations were grouped into analytically relevant exposure categories, including fieldwork, informal commerce, high physical workload, low physical workload, and long work hours. A reproducible mapping scheme is provided in **Supplementary Table S1**. Occupational exposure classification was based exclusively on documentation recorded prior to the diagnosis of preeclampsia to minimize outcome-dependent documentation bias.

**Antenatal access variables**

Antenatal access was assessed using three indicators: total number of antenatal care visits, estimated geographic accessibility to the nearest health facility, and reported use of traditional medicine as a proxy for limited access to allopathic care [10,11].

**Obstetric and medical history variables**

Obstetric and medical history variables included nulliparity, personal history of preeclampsia, chronic hypertension, gestational diabetes mellitus, urinary tract infection during pregnancy, and family history of hypertension among first-degree relatives [12–15]. Personal history of preeclampsia and chronic hypertension were retained as key exposure variables due to their strong and well-documented association with severe and superimposed preeclampsia.

**Nutritional status**

Nutritional status was assessed primarily using body mass index (BMI). Obesity was defined as a BMI ≥30 kg/m² based on height and weight recorded during prenatal care. When anthropometric data were incomplete, a documented clinical diagnosis of obesity was used. Explicit clinical judgment without anthropometric support was applied only when clearly stated in the medical record [4,35]. Sensitivity analyses excluding women classified solely by clinical judgment yielded similar effect estimates, indicating that potential misclassification did not materially affect the observed associations.

**Psychosocial variables**

Prior psychosocial stress was defined as documentation of at least one relevant psychosocial condition occurring before the diagnosis of preeclampsia. These included a documented history of anxiety or depression, exposure to adverse life events during pregnancy such as bereavement, domestic violence, or marital separation, and clinical notes indicating persistent emotional distress, psychological overload, or significant stress [29–31].

**Perinatal outcomes**

Perinatal outcomes included mode of delivery, gestational age at delivery, neonatal birth weight, and early neonatal complications such as a one-minute Apgar score <7, admission to the neonatal intensive care unit, or respiratory complications. Other clinically relevant outcomes, including eclampsia, HELLP syndrome, acute pulmonary edema, maternal or neonatal death, and use of magnesium sulfate, were not consistently documented in the available medical records and therefore could not be reliably analyzed [33, 36–38]. These outcomes were reported descriptively and were not treated as predictors in multivariable analyses.
