# Supplementary material for: Clinical, social, and occupational determinants of severe preeclampsia: a multifactorial case–control study on maternal health inequities in Peru
Source: BMC Pregnancy Childbirth. 2026 Jan 21;26:297. doi: 10.1186/s12884-026-08653-w (PMC12998109; doi:10.1186/s12884-026-08653-w)
Supplement: Supplementary file 2 — Supplementary Material 2. [file 12884_2026_8653_MOESM2_ESM.docx]

**Supplementary Table S1. Mapping of occupational categories using CNO‑Perú and ISCO‑08**

| **Recorded occupation (clinical chart)** | **CNO‑Perú code** | **ISCO‑08 major group** | **Analytical category used in the study** |
| --- | --- | --- | --- |
| Farmer, agricultural worker, field laborer | 6111 / 9211 | Group 6 (Skilled agricultural workers) / Group 9 (Elementary occupations) | **Fieldwork** |
| Street vendor, informal seller, market vendor | 5221 / 5244 | Group 5 (Service and sales workers) | **Informal commerce** |
| Domestic worker, cleaning services | 5153 | Group 5 (Service and sales workers) | **High physical workload** |
| Factory worker, warehouse assistant, manual laborer | 9321 / 9333 | Group 9 (Elementary occupations) | **High physical workload** |
| Office assistant, secretary, administrative staff | 4110 | Group 4 (Clerical support workers) | **Low physical workload (reference)** |
| Student, homemaker | Not applicable | Not applicable | **Non‑employed / baseline** |
| Any occupation ≥40 hours/week | — | — | **Long work hours (>40 h/week)** |
