# Supplementary material for: Clinical, social, and occupational determinants of severe preeclampsia: a multifactorial case–control study on maternal health inequities in Peru
Source: BMC Pregnancy Childbirth. 2026 Jan 21;26:297. doi: 10.1186/s12884-026-08653-w (PMC12998109; doi:10.1186/s12884-026-08653-w)
Supplement: Supplementary file 3 — Supplementary Material 3. [file 12884_2026_8653_MOESM3_ESM.docx]

## **Supplementary Table S2. Obstetric and medical history of participants**

| **Variable** | **Cases (n = 237)** | **Controls (n = 483)** | **Total (n = 720)** | **p-value** |
| --- | --- | --- | --- | --- |
| Nulliparity | 115 (48.5%) | 122 (25.3%) | 237 (32.9%) | <0.001 |
| History of preeclampsia | 53 (22.4%) | 15 (3.1%) | 68 (9.4%) | <0.001 |
| Chronic hypertension | 47 (19.8%) | 31 (6.4%) | 78 (10.8%) | <0.001 |
| Gestational diabetes | 27 (11.4%) | 23 (4.8%) | 50 (6.9%) | 0.001 |
| Urinary tract infection | 38 (16.0%) | 45 (9.3%) | 83 (11.5%) | 0.009 |
| Family history of hypertension | 46 (19.4%) | 49 (10.1%) | 95 (13.2%) | <0.001 |

Table footnote: Univariate comparisons were performed using Pearson’s Chi-square test. Results are presented as absolute frequencies (n) and percentages (%). Statistical significance was set at p < 0.05.
