# Supplementary material for: Clinical, social, and occupational determinants of severe preeclampsia: a multifactorial case–control study on maternal health inequities in Peru
Source: BMC Pregnancy Childbirth. 2026 Jan 21;26:297. doi: 10.1186/s12884-026-08653-w (PMC12998109; doi:10.1186/s12884-026-08653-w)
Supplement: Supplementary file 4 — Supplementary Material 4. [file 12884_2026_8653_MOESM4_ESM.docx]

**Supplementary Table S3. Perinatal outcomes of participants**

| **Variable** | **Cases (n = 237)** | **Controls (n = 483)** | **Total (n = 720)** | **p-value** |
| --- | --- | --- | --- | --- |
| Cesarean delivery | 162 (68.4%) | 157 (32.5%) | 319 (44.3%) | <0.001 |
| Preterm birth (<37 weeks) | 100 (42.2%) | 47 (9.7%) | 147 (20.4%) | <0.001 |
| Neonatal weight <2500 g | 90 (38.0%) | 40 (8.3%) | 130 (18.1%) | <0.001 |
| Adverse neonatal outcome | 52 (21.9%) | 27 (5.6%) | 79 (11.0%) | <0.001 |

Table footnote: Univariate analysis was performed using Pearson’s Chi-square test to compare proportions between groups. Statistical significance was set at p < 0.05.
